# Quality of Life Among Head and Neck Cancer Patients at a Tertiary Care Hospital in Assam, India: A Mixed-Methods Study

**DOI:** 10.7759/cureus.100664

**Published:** 2026-01-03

**Authors:** Ashfia Habib, Kumaril Goswami, Jutika Ojah, Mridul Kumar Sarma, Achyut Chandra Baishya

**Affiliations:** 1 Department of Community Medicine, Gauhati Medical College and Hospital, Guwahati, IND; 2 Department of Head and Neck Oncology, State Cancer Institute, Guwahati, IND

**Keywords:** assam, cancer of the head and neck, (eortc)-qlq-c30, mixed method study, quality-of-life

## Abstract

Introduction: The physical, psychological, and social functioning of patients with head and neck cancer (HNC) is profoundly affected, resulting in considerable challenges to their quality of life (QoL). This mixed-method study used both quantitative and qualitative methods to evaluate the QoL of HNC patients who were attending a tertiary cancer centre in Assam.

Materials and methods: Eighty adult HNC patients who were seen in the State Cancer Institute's outpatient department in Guwahati participated in a cross-sectional mixed-method study. Quantitative data were collected using the Assamese versions of the European Organisation for Research and Treatment of Cancer Quality of Life Questionnaire - Core 30 (EORTC QLQ-C30) and European Organisation for Research and Treatment of Cancer Quality of Life Questionnaire - Head and Neck 43 (EORTC QLQ-H&N43) questionnaires. Relationships between the functional and symptom domains and overall QoL were investigated using correlation analysis. To explore patients’ experiences related to physical, emotional, social, and role functioning, semi-structured interviews were conducted. Integration of quantitative and qualitative findings ensured methodological rigour.

Results: While emotional and role functioning were significantly compromised, cognitive functioning displayed the highest mean functional score. Emotional (45.3%) and role functioning (54.1%) showed lower scores, while physical (64.4%) and social functioning (52.6%) demonstrated moderate limitation. Cognitive functioning was the highest-scoring domain (75.3%). The most affected symptom domains included fatigue (56.2%), financial difficulty (55.2%), dry mouth (55.9%), sticky saliva (53.2%), social contact problems (53.7%), appetite loss (44.8%), and pain (41.3%). The H&N43's recently measured domains of sexual desire, neck/skin symptoms, and fear of recurrence also demonstrated a moderate impact. Persistent pain, difficulty swallowing, reliance on food, speech impairment, social disengagement due to disfigurement, and severe emotional distress were all highlighted in the qualitative findings. Many patients suffered from financial toxicity, strained family ties, and diminished work capacity.

Conclusion: In the social, emotional, physical, and financial spheres, HNC substantially lowers QoL. When paired with psychological distress and financial stress, symptom burden, particularly xerostomia, swallowing issues, and exhaustion, strongly predicts lower overall QoL. Improving survivorship outcomes requires comprehensive multidisciplinary care that addresses financial counselling, psychological support, and symptom management.

## Introduction

In any organ system, cancer is a disease that results in unchecked cell growth. People of different ages, genders, nationalities, ethnicities, socioeconomic backgrounds, educational attainment, and geographic locations are all impacted. It may manifest as chronic (long-term), sub-acute (slow-onset), or acute (sudden onset). In addition to more serious symptoms like bleeding, obstruction, and growths, it can also cause less specific ones like fever, diarrhoea, or weight loss. As a disease, it can keep people from realising their full potential in terms of their bodies, minds, and finances. Patients, their families, physicians, healthcare providers, and taxpayers are all very concerned about this [1]. With nearly 10 million deaths from the disease in 2020, cancer is the leading cause of death globally. Nearly one-third of all cancer deaths are caused by tobacco use, high body mass index, alcohol use, a diet low in fruits and vegetables, and inactivity. In low- and lower-middle-income countries (LMICs), more than 30% of cancer cases are caused by infections that cause cancer, such as hepatitis and HPV [2].

According to the Global Cancer Observatory (GLOBOCAN), there will be 19.3 million incident cases of cancer globally in 2020. India came in third place, behind the US and China. According to GLOBOCON, the number of cancer cases in India would reach 2.08 million, representing a 57.5% increase from 2020 to 2040 [3]. By 2025, the country's cancer case count is expected to rise by 12%, according to the Indian Council of Medical Research-National Centre for Disease Informatics and Research (ICMR-NCDIR) National Cancer Registry Programme [4]. The squamous cells lining the mucosal surfaces of the head and neck are usually where cancers classified as HNCs begin to grow. Squamous cell carcinomas of the head and neck are the name given to these tumours. Although they are much less common than squamous cell carcinomas, HNCs can also originate in the salivary glands, sinuses, or muscles or nerves in the head and neck [5]. HNCs can develop in the paranasal sinuses, nasal cavity, salivary glands, throat (pharynx), voice box (larynx), and oral cavity.

A multifaceted concept, quality of life (QoL) includes aspects related to mental, emotional, social, and physical functioning [6]. A related concept of QoL, well-being, assesses the positive elements of an individual's life, including happiness and contentment [7]. In oncology, where life expectancy is limited, and treatment rarely results in full recovery, the idea of QoL has gained significance in patient care. QoL and survival are the main objectives for HNC [8]. Health-related quality of life (HRQoL) is especially relevant in HNC, as the disease and its treatment often affect key functions such as speech, swallowing, breathing, and facial appearance, which are closely linked to social interaction and psychological well-being. This is due to the potential for visibly crippling physical issues as well as the psychological repercussions of altered body image and diminished function. In addition to having a potentially fatal illness, patients with HNC must cope with the effects of treatment on many facets of their QoL, such as functional impairments related to speech, swallowing, hearing, and breathing that are vital to social interaction [9].

According to the World Health Organisation (WHO), QOL is "an individual's perception of their position in life in relation to their goals, expectations, standards, and concerns as well as in the context of the culture and value systems in which they live" [10]. According to the most recent report of the National Cancer Registry Programme (ICMR-NCDIR), which analysed data from 43 Population-Based Cancer Registries between 2015 and 2019, the North-Eastern states recorded the highest overall cancer incidence in the country. Districts such as Aizawl, East Khasi Hills, Papumpare, and Kamrup (Urban) ranked among the highest. HNCs also showed a particularly high burden in this region, with East Khasi Hills, Kamrup (Urban), Meghalaya, and Nagaland reporting some of the highest incidence rates nationally [11]. Therefore, the present study aimed to assess the HRQoL among patients with HNC attending a tertiary cancer centre in North-East India using the European Organisation for Research and Treatment of Cancer Quality of Life Questionnaire - Core 30 (EORTC QLQ-C30) and European Organisation for Research and Treatment of Cancer Quality of Life Questionnaire - Head and Neck 43 (EORTC QLQ-H&N43) questionnaires. In addition, the study sought to explore the lived experiences of these patients through qualitative interviews to complement and contextualise the quantitative findings.

## Materials and methods

This cross-sectional mixed-method study was conducted at the State Cancer Institute in Guwahati, Assam, India, over a three-month period from July to September 2024. Ethical approval was obtained from the Institutional Ethics Committee of the State Cancer Institute, Guwahati (Approval No: SCI/GMC/ECR/2020/156), and written informed consent was obtained from all participants before recruitment. Adult patients aged 18 years and above, with no upper age limit, who had been receiving active treatment for HNC within the previous two years, were purposively selected as the study population. “Active treatment” was defined as current or recent receipt (within two years) of surgery, radiotherapy, chemotherapy, immunotherapy, or combination therapy for HNC. Patients with terminal illness were excluded if their clinical or emotional condition limited their ability to participate in interviews.

A total of 80 participants were recruited using non-probability purposive sampling. The sample size was determined pragmatically based on outpatient attendance volume during the study period and was considered sufficient to meet study objectives, while allowing thematic saturation for the qualitative component. The study used a mixed methods approach to collect data on patients' QoL, and quantitative data were obtained using the Assamese version of the EORTC QLQ, both generic and head-and-neck specific. The data collection phase lasted one month, during which all eligible patients attending the Head and Neck Oncology Department of the State Cancer Institute were recruited. The basic questionnaire (EORTC QLQC30) applies to all cancer patients, whereas the disease-specific questionnaire (EORTC QLQ-H and N43) is tailored to patients with cancer in the head and neck regions. These devices measure patients' symptoms and functional condition, including the nature of their physical ailments.

EORTC QLQC-30 assesses five functional scales: physical functioning, role functioning, emotional functioning, cognitive functioning, and social functioning. The scale includes three symptom scales and six individual items. A high score on a symptom scale or item indicates a high severity of symptoms. The EORTC QLQ-C30 uses a seven-point Likert scale and two questions based on self-evaluation of health to evaluate patients' global QOL. While the symptom scales ranged from 0.5553 to 0.8296, the EORTC QLQ-C30 functional scales (apart from cognitive functioning) and global QLQ had internal reliability coefficients (Cronbach's alpha) ranging from 0.8025 to 0.9431. The reliability coefficient for cognitive functioning was low (0.6341) and considerably worse in the previous version of the test (version 2) [12].

The EORTC QLQ-H&N43 is an HRQoL questionnaire of 12 multi-item and seven single-item scales designed specifically for patients with HNC to assess symptoms and functional issues. It measures problems with teeth, pain, swallowing, senses, body image, social eating, and more. Raw scores are converted into a scale of 0 to 100, where 100 indicates a worse HRQoL or a higher symptom burden.

To investigate the relationship between variables and their effect on patient QoL, quantitative data were analysed using Julius AI (Julius AI Inc., San Francisco, CA, USA), an artificial intelligence-assisted analytical platform that supports statistical computation, text analysis, and data visualisation. The software was used to assist in organising and exploring the data; all outputs were cross-checked and interpreted manually by the investigators to ensure accuracy. For the qualitative component, semi-structured in-depth interviews were conducted with a subset of the participating HNC patients. Interviews were held in a private counselling room within the State Cancer Institute to ensure comfort and confidentiality. Each interview lasted approximately 30-45 minutes and followed an interview guide covering physical symptoms, emotional well-being, social functioning, role functioning, and financial concerns. Audio-recording was not performed; instead, detailed field notes were taken during the interview and expanded immediately afterwards. The notes were anonymised and reviewed for completeness before analysis. Thematic analysis was undertaken, and coding was carried out manually by the research team to identify recurring patterns and overarching themes.

## Results

Results from quantitative variables

**Figure 1:**
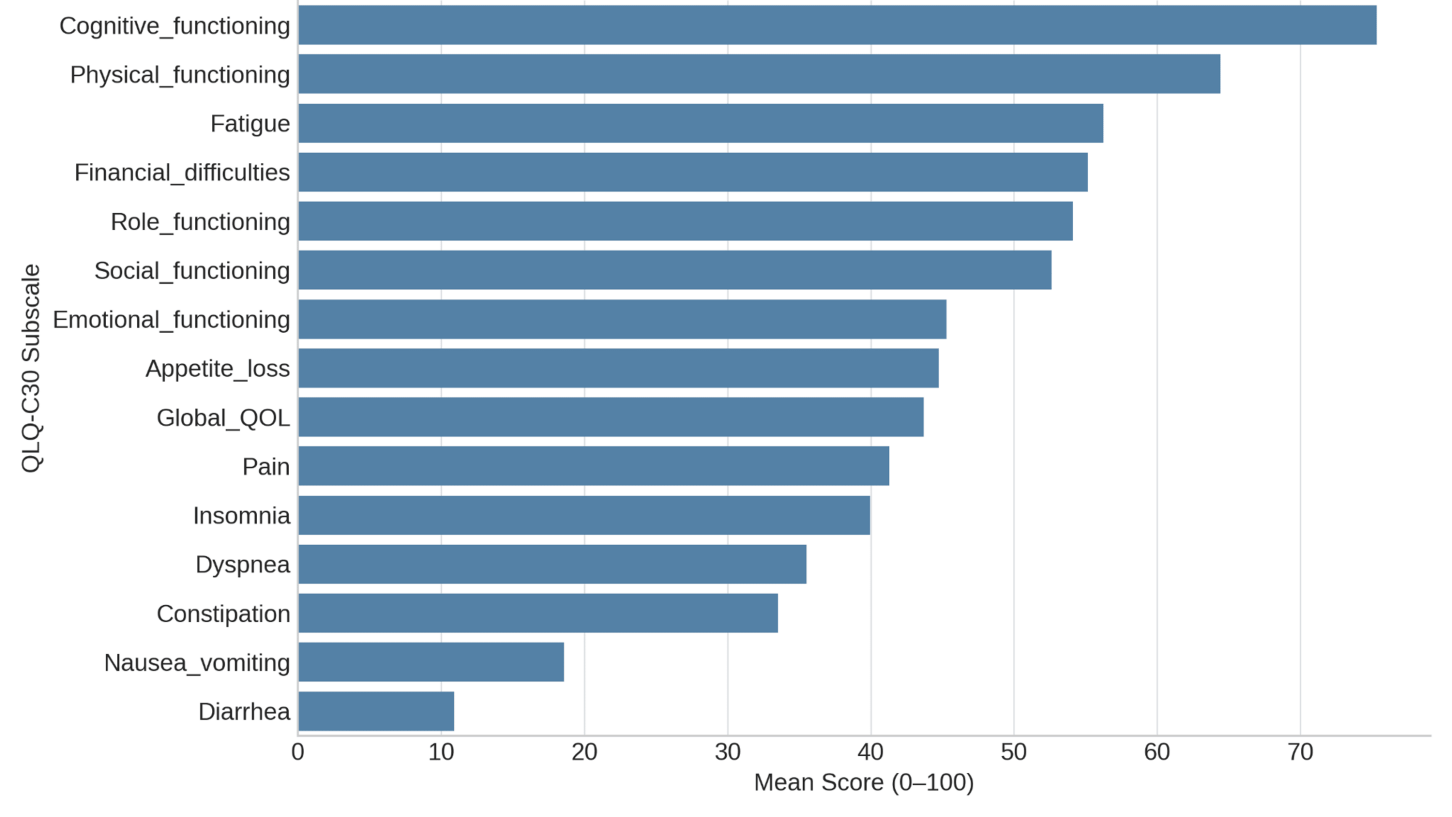
Mean Scores of the EORTC QLQ-C30 functional and symptom scales. Higher functional scores indicate better functioning, while higher symptom scores indicate greater symptom severity. EORTC QLQ-C30: European Organisation for Research and Treatment of Cancer Quality of Life Questionnaire-Core 30; QOL: quality of life

**Figure 2:**
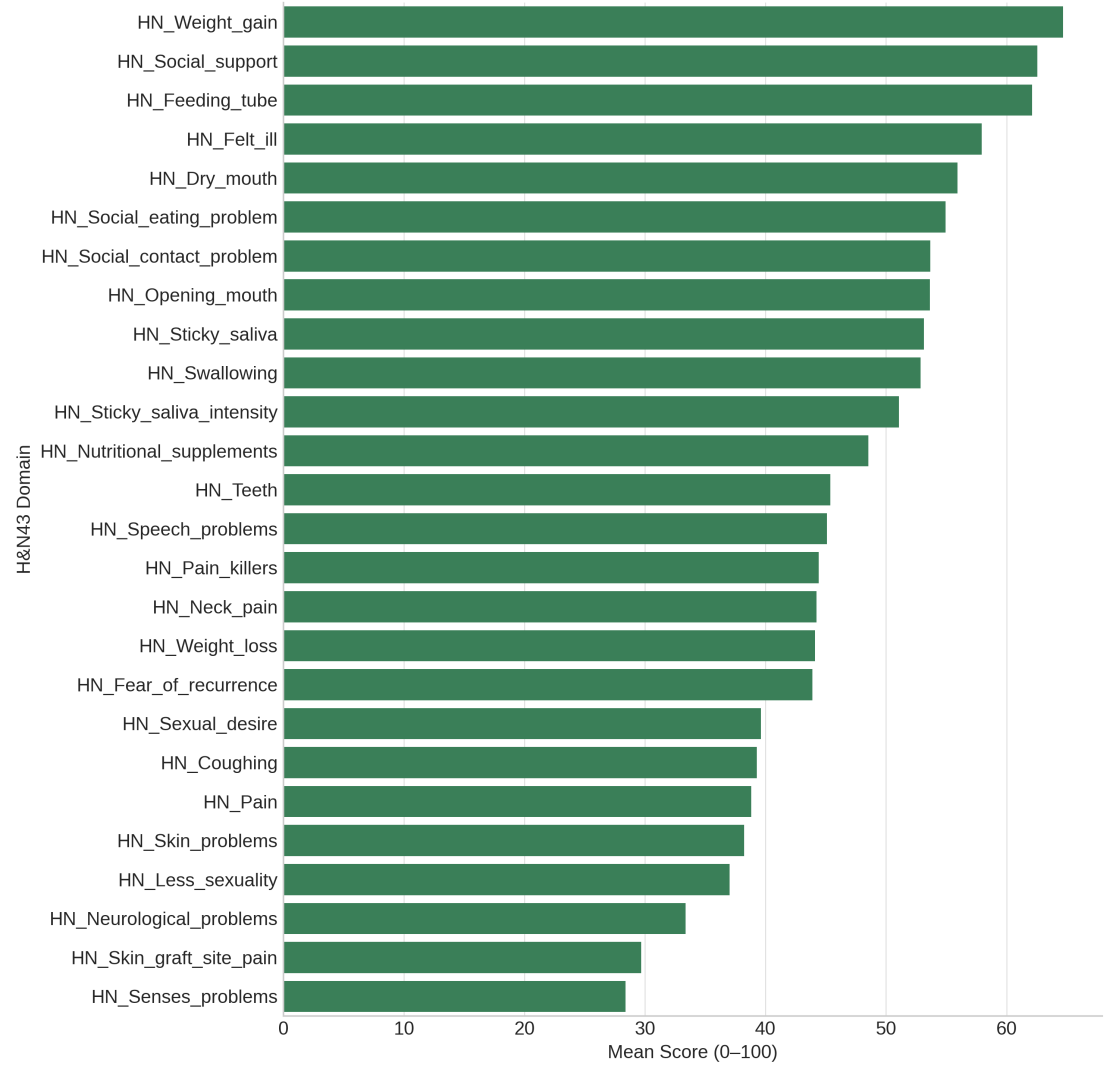
Mean Scores of the EORTC QLQ-H&N43 symptom and function scales. Swallowing, dry mouth, and saliva-related issues remain the most affected domains, while emotional aspects like fear of recurrence also contribute substantially to reduced quality of life. EORTC QLQ-H&N43: European Organisation for Research and Treatment of Cancer Quality of Life Questionnaire-Head and Neck 43; HN: head and neck

All cancer patients can use the EORTC QLQ-C30 to assess general QoL domains. The mean scale scores of the 90 participants showed trends that were in line with previous research. The highest score (mean = 75.3) was for cognitive functioning, indicating retained memory and mental clarity. While emotional and role functioning were lower (means 45.3 and 54.1), physical and social functioning showed moderate impairment (means 64.4 and 52.6, respectively), suggesting a substantial psychosocial burden. Symptoms of treatment-related distress included moderate fatigue (mean = 56.2), pain (mean = 41.3), and appetite loss (mean = 44.8) as mentioned in Figure 1. Financial difficulties were considerable (mean = 55.2), demonstrating economic strain linked to cancer care. The EORTC QLQ-H&N43, an updated version of the H&N35 module, captures head-and-neck cancer-specific symptoms. Prominent issues included swallowing difficulties, dry mouth, sticky saliva, and problems with social eating and contact - all with mean scores above 50, highlighting significant post-treatment morbidity. Newly added scales - fear of recurrence, neck pain, skin problems, neurological symptoms, and sexual desire - also showed moderate impact (means 30-50) as mentioned in Figure 2.

Correlation Analysis

**Figure 3:**
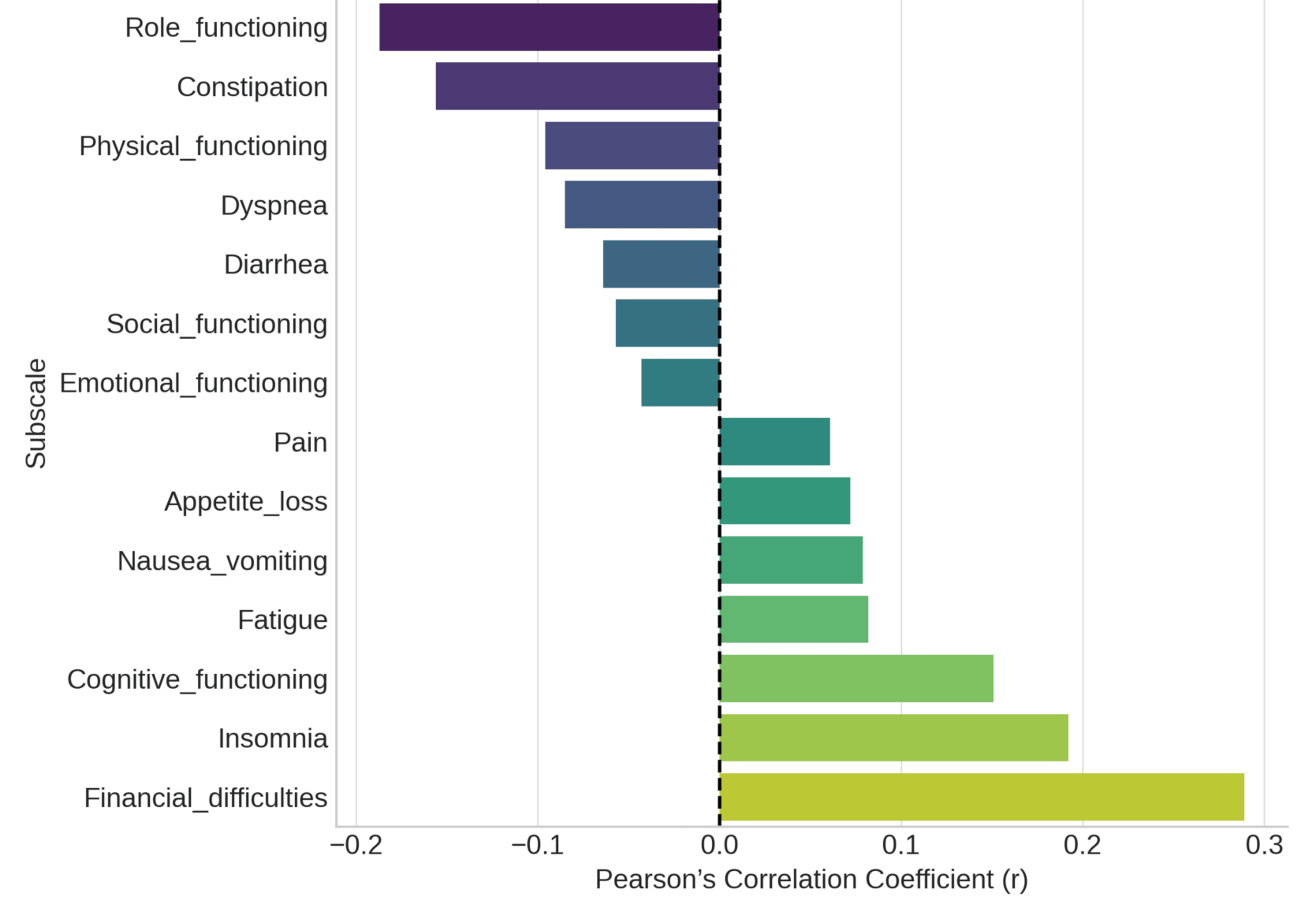
Correlation of EORTC QLQ-30 subscales with global quality. EORTC QLQ-30: European Organisation for Research and Treatment of Cancer Quality of Life Questionnaire-Core 30

To assess the connections between each subscale and the overall QoL score, Pearson's correlation coefficients were calculated. Insomnia (r = 0.19) and financial difficulties (r = 0.29) demonstrated the strongest positive correlations with lower overall QOL, suggesting that sleep issues and economic stress have a significant impact on perceived well-being. Weak negative correlations between role functioning and constipation were found (r = -0.19 and -0.16), highlighting the multifaceted effects of illness on QoL. The overall correlation matrix supported the predicted pattern, showing that while functional scales had a negative correlation with symptom burden, symptom scales had a positive correlation, as mentioned in Figure 3.

Demographic Correlation

**Figure 4:**
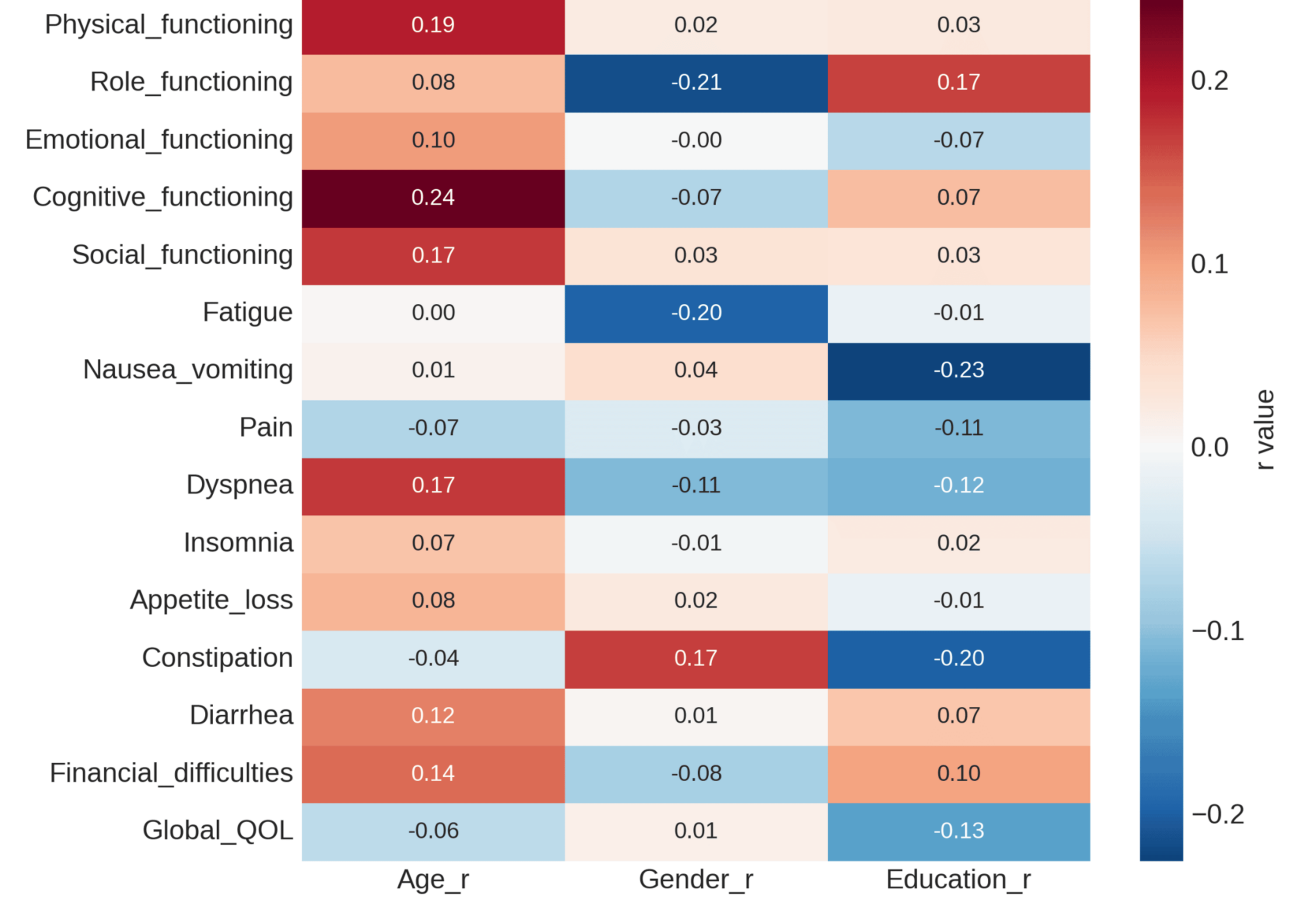
Correlation of demographic variables with EORTC QLQ-C30 subscales. EORTC QLQ-C30: European Organisation for Research and Treatment of Cancer Quality of Life Questionnaire-Core 30; QOL: quality of life

There were weak to moderate correlations between the QOL domains and demographic characteristics (age, gender, and education). While gender and education had little bearing, older age was marginally associated with decreased physical functioning (r = 0.19). Reduced gastrointestinal symptoms (such as constipation, r = -0.20) were marginally associated with higher education. This pattern implies that the effects of illness and treatment have a greater influence on QOL outcomes than demographic factors, as mentioned in Figure 4. Physical and psychological constraints are the main causes of the moderate global QoL. New EORTC QLQ-H&N43 scales have been added to improve understanding of current patient experiences, especially with regard to social support, sexual health, and recurrence fear. These revelations highlight the value of all-encompassing rehabilitation, which includes social, psychological, and nutritional therapies, in order to improve QoL following treatment.

Results from the qualitative interview

Physical Operation

Patients frequently reported a variety of pain-related symptoms during in-depth interviews at the State Cancer Institute, including pain in the stomach, throat, joints, shoulders, chest, and surgical sites. The most common causes of dyspnoea, or difficulty breathing, were nasal blockage, cold sensations during sleep, or the tracheotomy procedure itself. For many patients, the primary causes of insomnia and sleep disturbances were chronic pain and nighttime urination. While several patients required sleep-inducing medications, one participant reported using alcohol occasionally to help initiate sleep. There were a lot of physical limitations. Activities of daily living (ADLs), such as eating, dressing, bathing, and using the restroom, required assistance for many patients. Additionally, there were obvious indications of nutritional problems; some patients were completely reliant on liquid or tube feeding and were unable to chew or swallow solid food, making them susceptible to carers. Patients frequently reported losing or altering their sense of taste, particularly for foods like sugar or ginger, while citric or spicy foods were difficult to tolerate. Some people had difficulty swallowing solid foods and needed liquids to aid in their chewing. Eating in public was often avoided due to the potential for food spills or embarrassing facial deformities.

One patient described, “I no longer eat in the same way. I prefer to eat by myself and am hesitant to eat in social situations. I now mostly rely on a liquid diet to survive.”

Another shared: “I need someone to be around all the time because I am completely dependent on others for my food, which must be administered via a feeding tube.”

Role and Cognitive Functioning

The majority of patients stated that their capacity to work effectively and physically had significantly decreased. Many were unable to return to work at all or were on long leaves of absence.

One person revealed, “I need help with my everyday tasks because I get tired easily now. I had to take a lengthy leave of absence due to a decline in my work performance.”

Patients also reported losing interest in hobbies and recreational activities. It was challenging to read newspapers or watch television due to poor focus and mental tiredness, which indicated impaired cognitive functioning.

Psychological and Emotional Processes

Emotional distress was one of the participants' primary themes. Particularly in relation to speech abnormalities, family burden, financial difficulties, and facial disfigurement, patients reported feelings of sadness, agitation, anxiety, and fear. The sense of uncertainty about the prognosis and recurrence increased emotional strain.

One patient described, “I consider my illness and my family's future all the time. I get depressed and angry sometimes.”

Patients reported avoiding social situations due to embarrassment or persistent enquiries about their condition. The social stigma attached to cancer, particularly in relation to obvious facial abnormalities, made isolation worse. Some claimed that prolonged hospital stays or time spent away from home for treatment had strained family relationships.

One participant stated: “I am worried about how I look. People keep asking questions, so I cover my face before going out.”

Another added, “I avoid social interaction because of my disfigured face and distorted speech. It is frustrating when people keep asking about my condition.”

Communication and Speech Issues

Patients who underwent radiation therapy or head and neck surgery reported speech distortion, difficulty speaking, and partial or complete voice loss. These issues hindered professional and social relationships.

A lawyer shared his experience: “My speech has become distorted. I can’t argue in court like before; even the judge asks me to repeat myself.”

Speaking on the phone or in public was challenging due to common articulation problems, dry mouth, and low voice pitch, which also increased social anxiety.

Social and Sexual Functioning

Participants also reported a decrease in intimacy and sexual desire with partners, often due to prolonged hospital stays and physical exhaustion. As a result of the illness, some people lost interest in life and were less likely to engage in social or recreational activities. Nonetheless, some respondents did discuss helpful coping mechanisms. They discussed cultivating deeper spiritual inclination, emotional resilience, and philosophical contemplation. Their experience with illness helped them develop self-awareness, acceptance, and patience.

One patient expressed: “I've grown more serene and spiritual. My outlook on life has altered as a result of this illness.”

**Figure 5:**
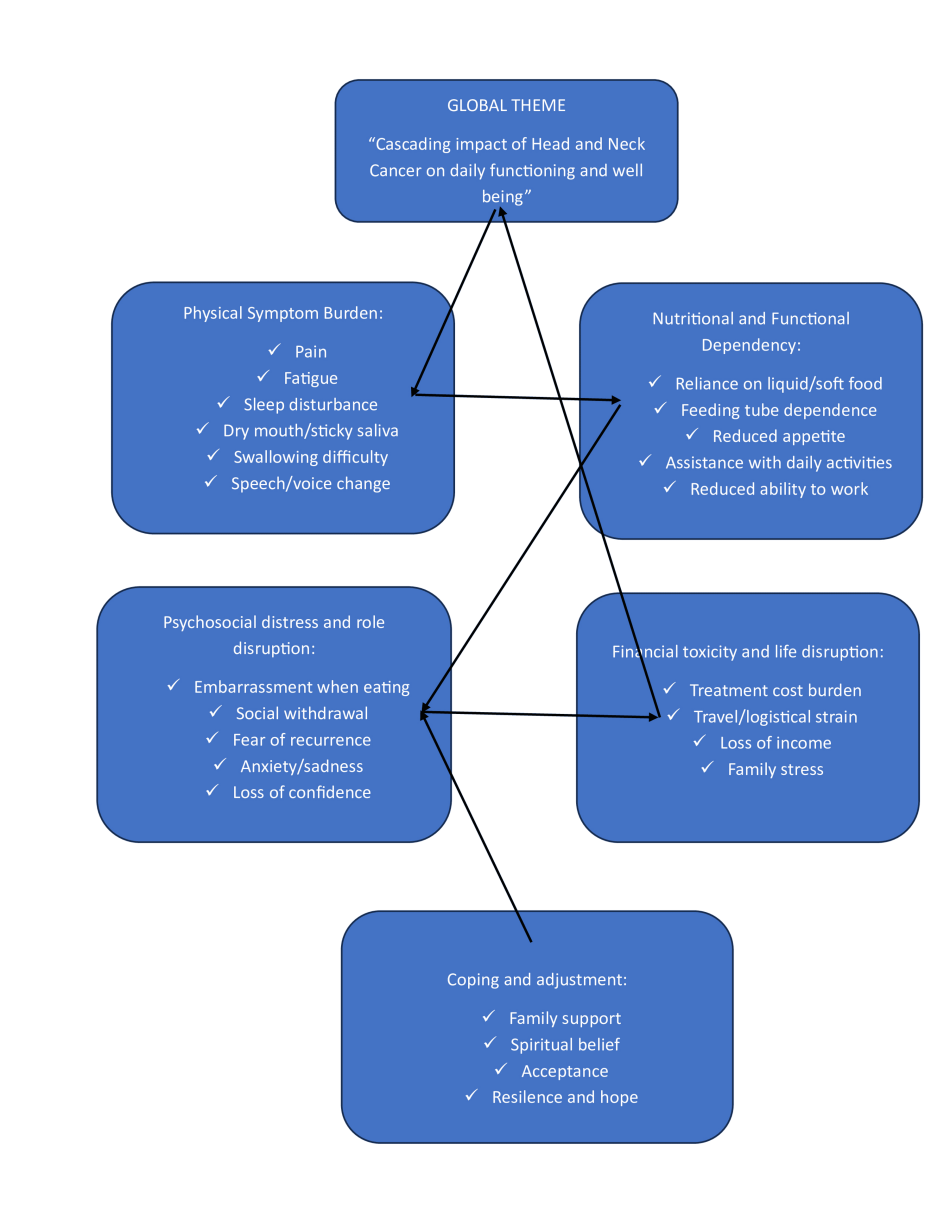
Thematic network illustrating how physical symptoms in head and neck cancer contribute to nutritional and functional dependency, psychosocial distress, and financial toxicity, ultimately reducing quality of life, with coping and support mechanisms acting as moderating influences.

The thematic network diagram, as mentioned in Figure 5, illustrates how HNC produces a cascading impact on patients’ daily functioning and overall well-being. Physical symptoms such as pain, fatigue, sleep disturbance, dry or sticky mouth, swallowing difficulty, and speech or voice changes form the starting point of this pathway. These problems often lead to nutritional and functional dependency, including reliance on liquid or soft diets, feeding assistance, and reduced capacity to work or perform daily activities. As independence declines, many patients experience psychosocial distress in the form of embarrassment, social withdrawal, anxiety, fear of recurrence, and loss of confidence, alongside financial toxicity arising from treatment costs, travel burden, and loss of income. Together, these interrelated effects substantially diminish the QoL. Coping mechanisms such as family support, spirituality, acceptance, and resilience act as moderating influences that help patients adapt but do not fully eliminate the burden of illness.

## Discussion

The current study's results show that cognitive domains had the highest functioning scores, whereas emotional and role functioning lagged considerably, indicating a disproportionate psychosocial burden among patients. Treatment-related side effects include fatigue, pain, dry mouth, difficulty swallowing, and decreased social eating and contact. Furthermore, insomnia and financial toxicity showed significant associations with overall QoL. When compared to symptom burden and functional impairment, demographic factors (age, gender, and education) seemed to have less of an impact. In a number of ways, these findings are in line with recent research.

First, the EORTC QLQ-H&N43 (Phase IV) international validation, which involved 812 patients worldwide, confirmed strong discriminant validity, good internal consistency (Cronbach's alpha >0.70 in most scales), and sensitivity to change in almost all symptom and functional domains, particularly swallowing, oral pain, social eating, and dental issues, which were also among the most impacted items in our data [13]. The scope of patient-reported outcomes is expanded by the inclusion of more recent domains in H&N43, such as fear of recurrence and neurological symptoms. Our moderate scores in these domains are consistent with the validation study's findings that these are important for differentiating known groups and identifying change [13]. Among HNC survivors, psychological distress is still widespread. Nearly half of patients experienced clinically significant anxiety or depression within two years of treatment, according to a 2024 multicenter European study. Both conditions are strongly linked to a lower global QoL. A high degree of distress was displayed by 41% of patients. Patients who had a tracheotomy or had previously had cancer in a different district were more distressed. After controlling for stage and the amount of time since the end of treatment, QoL was consistent across treatment types, with the exception of patients receiving combined therapy experiencing higher levels of suffering from sensory issues, social eating, and dry mouth [14].

Financial toxicity is becoming more and more recognised as a crucial factor influencing HNC patients' QoL. In a recent cross-sectional survey conducted in Australia, Smith et al. (2023) measured out-of-pocket expenses and discovered strong negative correlations between financial toxicity and HRQoL among survivors of HNC one to three years after radiation therapy [15]. Similarly, a study by Rosi-Schumacher et al. (2023) found that financial burden is consistently associated with worse QoL, higher symptom burden, and worse psychosocial outcomes. It also revealed direct and indirect costs as well as measurement tools across multiple studies [16]. These results support our finding that there is a significant relationship between financial hardship and overall QoL. Third, recent randomised and observational studies have provided strong evidence for symptom domains like xerostomia (dry mouth), salivation issues, difficulty swallowing, sticky saliva, and associated oral functional decline. Thirty randomised controlled trials (RCTs) were included in the network meta-analysis of non-pharmacological interventions for radiotherapy-induced xerostomia (2025), which confirmed that, when compared to standard care, interventions like mouthwash and gel improved xerostomia symptoms and xerostomia-related QoL [17]. Additionally, stimulated salivary flow and xerostomia gradually improved over a five-year period in a long-term follow-up study of patients receiving intensity-modulated radiation therapy (IMRT), although patients with higher radiation doses recovered more slowly [18]. These corroborate the high symptom mean scores for salivation and dry mouth in our data and point to the necessity of ongoing observation and treatment. Our correlation analyses also support new findings. Sleep disorders and insomnia are becoming more widely acknowledged as independent indicators of lower overall QoL in HNC survivors [19]. Studies linking financial toxicity to treatment non-adherence, increased distress, and poorer HRQoL are consistent with the strong correlation observed between financial difficulties and overall QoL in our study [15,16]. Our data revealed weak to moderate correlations between demographic factors, indicating that the QoL landscape is dominated by functional loss and underlying symptom severity. While sociodemographic risk factors (income, insurance status, education) are significant, they frequently act through mediating variables like treatment modality, stage, and symptom burden rather than independently, according to recent survivorship consensus statements [20].

Clinical implications

These results have a number of ramifications. First, it is recommended that comprehensive QoL instruments such as QLQ-H&N43 be used on a regular basis. These instruments capture not only more recent areas like fear of recurrence, neurological symptoms, and sexual function, but also more established domains like pain, swallowing, and oral symptoms. Second, patients with lower incomes, no insurance, or high out-of-pocket costs may need early social and financial support; therefore, financial toxicity should be screened on a regular basis. Third, it is critical to implement interventions that address swallowing, xerostomia, and other oral functional impairments. Survivorship care plans should incorporate non-pharmacological interventions that have demonstrated efficacy, such as oral moisturisers and special mouthwashes. Fourth, psychosocial support, including peer support, counselling, and sleep interventions, is essential for emotional and role functioning.

Limitations

The cross-sectional method restricts the ability to determine causality. Further empirical data is necessary for generalisability to LMICs, as many recent studies (including those used for citations) are carried out in high-income settings.

Future directions

Longitudinal cohort studies employing QLQ-H&N43 to monitor domain trajectories from diagnosis through long-term survivorship should be the focus of future research. There is a need for intervention trials for high burden domains such as sleep, financial toxicity, swallowing, and xerostomia. It is crucial to develop implementation strategies in LMICs to guarantee access to symptom mitigation (financial aid, swallowing therapy, and saliva substitutes). Lastly, cost-effectiveness and policy decisions will benefit from the incorporation of health utility measures (e.g., the Head and Neck Cancer 8-Dimension (HNC-8D), recently developed based on HN43) [21].

## Conclusions

This study indicates that HNC significantly reduces QoL in several domains. Using the EORTC QLQ-C30 and QLQ-H&N43, we found that emotional functioning, dry mouth, fatigue, and swallowing were the most challenging, whereas cognitive function was mainly unaffected. Global QoL was found to be significantly influenced by overwhelming demographic factors, financial toxicity, and insomnia. The findings emphasise the importance of offering survivors comprehensive, multidisciplinary care that considers their physical, psychological, and socioeconomic needs. The QLQ-H&N43 can be routinely used to guide individualised symptom management and identify patients who require additional support. Future research should focus on longitudinal monitoring and targeted interventions to alleviate high-burden symptoms, even though policy efforts must prioritise reducing financial distress to improve long-term QoL.
